# Gamma knife radiosurgery for cavernous malformations: a comprehensive study on symptom relief, hemorrhage rates, and histopathological changes

**DOI:** 10.1007/s10143-025-03257-y

**Published:** 2025-01-30

**Authors:** Serkan Civlan, Berk Burak Berker, İlker Kiraz, Nagihan Yalçın, Ergin Sağtaş, Emrah Egemen, Barış Albuz, Nevzat Doğukan Erbek, Mehmet Erdal Coşkun, Ümit Akın Dere, Mustafa Kıvrak, Fatmanur Kaçmaz, Feridun Acar, Sait Şirin, Fatih Yakar

**Affiliations:** 1 Department of Neurosurgery, Pamukkale University School of Medicine, Kim Burchiel Gamma Knife Center, Denizli, Türkiye Turkey; 2 Department of Neurosurgery, Hatay Education and Research Hospital, Hatay, Türkiye Turkey; 3 Department of Pathology, Pamukkale University School of Medicine, Denizli, Türkiye Turkey; 4 Department of Radiology, Pamukkale University School of Medicine, Denizli, Türkiye Turkey; 5 Odak Hospital, Department of Neurosurgery, Denizli, Türkiye Turkey; 6 Department of Neurosurgery, Medicana International Ankara Hospital, Ankara, Türkiye Turkey

**Keywords:** Cavernoma, Cavernous malformation, Adverse radiation effects, Hemorrhage

## Abstract

This study aims to demonstrate the effect of gamma knife radiosurgery (GKRS) on symptoms, hemorrhage rates, and histopathological changes in patients with cavernous malformations (CMs), regardless of whether the symptomatic lesions are hemorrhagic. This single-center retrospective study evaluated symptomatic patients with single CMs treated with GKRS between 2016 and 2023. The patients’ demographic data, presenting symptoms, GKRS radiation dose, complications developed during follow-up (hemorrhage, radiotoxicity), the rate of symptom improvement, and histopathological changes of surgically removed CMs were recorded. Our study included 134 symptomatic patients, of whom 55 were male (41%) and 79 were female (59%). Among the patients, 80 (59.7%) had CMs in the lobar region, 23 (17.1%) in the cerebellum, 16 (12%) in the brainstem, and 15 (11.2%) in the basal nuclei/thalamus. The mean target volume was 0.64 ± 1.23 cm^3^, and the mean tumor margin dose was 14.89 ± 1.90 Gy. Symptoms were completely recovered in 95 patients (70.9%), while 18 (13.4%) experienced partial recovery. The annual hemorrhage rate (AHR) reduced from 6.26 to 1.01 following GKRS. Adverse radiation effects (ARE) were encountered in ten patients (7.46%) of cases, with only three patients (2.2%) being permanent. The mean follow-up period was 35.57 ± 23.54 months (2–83 months). Two patients without hemorrhage underwent surgical removal of CM due to symptomatic deterioration after GKRS. Histopathological examination demonstrated numerous vascular structures with luminal narrowing due to hyalinization and fibrinoid necrosis caused after GKRS. Our findings suggest that GKRS may help reduce hemorrhage rates and potentially relieve symptoms in patients with symptomatic CMs, although further long-term studies are necessary to confirm these observations and to fully assess potential risks. Since there is no radiological method to evaluate the impact of GKRS on CMs, our study examines the histopathological changes that occurred following the GKRS. The histopathological changes prove that GKRS alters the morphology of the CMs and thus can relieve symptoms and reduce hemorrhage rates associated with CMs.

## Introduction

CMs are a specific type of low-flow vascular lesions encountered in approximately 0.5% of the general population and represent 8–15% of all intracranial vascular lesions [1, 30, 39, 45]. These angiographically occult pathologies comprise sinusoidal vascular channels lined by an endothelial layer filled with different stages of stagnated blood without intervening brain parenchyma inside the lesion [35, 37, 48]. CMs may occur in both supratentorial and infratentorial regions, including the brainstem, with variations in size and formation [8, 18]. The AHR is estimated to be 0.25–16.5%, with increased hemorrhage rates observed after the first hemorrhage [9, 31, 39]. Besides patients represented with bleeding, there is a considerable number of patients experiencing neurological symptoms such as focal neurological deficits, seizures, imbalances, or headaches without radiologically documented hemorrhages [1, 35, 37, 48].

Various management strategies for symptomatic CMs have been described [2, 3]. Although total resection of a CM is considered the first-line treatment strategy, it carries considerable risk of mortality and morbidity, even with advances in microsurgical techniques, specifically for the lesions in deep-located or eloquent areas [13, 14, 17, 36]. Besides microsurgical interventions, previous studies demonstrate that GKRS is a safe and effective treatment method for CMs [25, 29, 32]. Since there is no imaging tool to confirm that a CM has been obliterated, the effect of GKRS on CM has been evaluated through clinical assessments in previous studies [26]. This study aims to demonstrate the effectiveness of GKRS in CMs through histopathological documentation, symptom relief, and reduction of hemorrhage rates in patients with CMs, regardless of whether the lesion has been hemorrhagic.

## Materials and methods

### Patient data and outcome assessment

A series of 134 symptomatic patients with single CM in various cerebral locations underwent the GKRS in a single center from 2016 to November 2023 and were retrospectively evaluated. To capture both short-term and long-term outcomes, patients were included in the study from the first day of GKRS treatment, regardless of the follow-up duration. This approach allowed for analyzing outcomes during the latent period following treatment.

A CM was considered symptomatic if it was associated with headache, seizure, neurological deficit, changes in the level of consciousness, imbalance, vomiting/nausea, regardless of whether radiologically documented hemorrhage following the exclusion of other diseases that may cause similar symptoms. Imbalance was defined as gait imbalance, vertigo, and/or dizziness, regardless of objective findings on neurological examination. A decrease in the level of consciousness was defined as at least a 1-point reduction in the Glasgow Coma Scale (GCS) from the patient’s baseline neurological status or disorientation.

Before initiating treatment, we thoroughly discussed all available options, with surgery being recommended as the primary choice. The treatment decision was made after evaluating the patient’s comorbidities, lesion location, hemorrhage status, and symptoms while also considering the patient’s preferences. Patients with comorbidities, lesions not abutting the pial surface on the brainstem, lesions located in profound and eloquent locations, and those who rejected surgery GKRS treatment were proposed.

For patients presenting with hemorrhages and a decrease in consciousness, GKRS was scheduled after an average waiting period of 6–8 weeks to allow hemorrhage resolution. During this period, these patients were admitted and closely monitored for neurological deterioration. After stabilization without signs of further deterioration, they were discharged with appropriate medications and prepared for GKRS.

CM diagnoses were established through evaluation by a five-year experienced neurosurgeon and a ten-year experienced neuroradiologist, along with consideration of CM’s characteristic Magnetic Resonance Imaging (MRI) features. Lesions were classified into four groups based on their location: lesions in the lobar region (frontal, temporal, parietal, and occipital), basal nuclei/thalamus, brainstem, and cerebellum. Radiological classification was made according to the Zabramski classification system [48]. Radiological features, bleeding, and coexistence with developmental venous anomaly (DVA) with CM were recorded.

The patients underwent GKRS with a radiologically identified hemorrhage, regardless of whether they were symptomatic. In the patients without hemorrhage, GKRS was applied only to symptomatic patients to decrease the AHR.

All patients underwent detailed neurological examinations at admission, after radiosurgery, discharge, and follow-up. Patient symptoms and history of microsurgical intervention before GKRS were evaluated. Patients were classified based on changes in their symptoms as “completely recovered,” “partially recovered,” “no changes,” or “worsened.” Seizure control rates were evaluated according to Engel’s classification [16]. In patients experiencing seizures, single or combination antiepileptic drugs were administered after evaluating any comorbidities. ARE were defined as perilesional edema in MRI and neurological signs or symptoms or worsening of preexisting deficits after the procedure.

Written informed consent was obtained from all patients. The study was conducted according to the ethical standards of the Declaration of Helsinki. Approval was granted by the institutional ethics committee.

### Radiosurgical technique

The GKRS was performed using the Leksell Gamma Knife Perfexion Model (Elekta AB, Stockholm, Sweden). After applying the Leksell stereotactic head frame under local anesthesia, high-resolution MRI and one-millimeter-sliced computerized tomography (CT) images were obtained. Contrast-enhanced T1 sequence MRI, T2 sequence MRI, Fluid Attenuated Inversion Recovery (FLAIR) sequence, and susceptibility-weighted imaging (SWI) sequences were used to determine the boundaries of lesions and hemosiderin rings. Lesion delineation was performed using the T2 sequence, and it was suggested that, since iron breakdown particles serve as potential radiation sensitizers, the target within the lesion should be located in the T2-defined hemosiderin-stained area rather than in the hemosiderin ring surrounding the lesion [9, 13, 24]. The margin of the CM was defined by the region with mixed signal change, excluding the surrounding hemosiderin ring and DVA if present.

Additionally, GKRS was performed after an average waiting period of 6–8 weeks in patients with hemorrhagic lesions to resolve the hemorrhage. All patients underwent a single GKRS session, including multiple isocenters and a 50% isodose line at the CM margin. After GKRS, all patients received 40 mg of intravenous methylprednisolone and were discharged within 3–24 h.

### Follow-up

Radiological and clinical follow-ups were started after the patient’s first admission with symptoms and continued at three-month intervals after treatment for the first year, six months for the second year, and annually after that. Pre-GKRS and post-GKRS AHRs were calculated by dividing the total number of hemorrhages by the total number of patients observed in the year.

### Statistical analysis

The relationship between the patient’s symptoms, CM features, and hemorrhage was assessed using the Mann-Whitney U and Pearson Chi-Square tests. Analyses were performed using SPSS version 15.0 for Windows (IBM Corporation, Chicago, Illinois, USA), with statistical significance set at *p* < 0.05. In addition, adjustments for multiple comparisons were made using the Bonferroni correction to enhance the robustness of the findings.

## Results

Of the 134 patients, 55 were male (41%) and 79 were female (59%). The mean age of the patients was 46.71 ± 15.86 years (range: 12–81 years). The mean follow-up period was 35.57 ± 23.54 months (2–83 months). Two patients had undergone previous subtotal surgical intervention, and one underwent GKRS at another institution. GKRS was performed for the second time for this patient due to rebleeding after 13 years of follow-up. All patients were symptomatic, and 54 patients (40%) had radiologically documented hemorrhages caused by CMs. Among these 54 patients, 51 had experienced a single hemorrhage, while 3 suffered multiple hemorrhages. According to Zabramski’s classification, 31 (23.1%) patients were categorized as grade 1, 96 (71.7%) as grade 2 and 7 (5.2%) as grade 3. Additionally, 77 (57.4%) CMs were found to coexist with a DVA. Ten patients (7.46%) had perilesional edema following GKRS, and DVA was present in six of them. There was no statistically significant relationship between the development of edema and the presence of DVA (*p* = 0.715). The mean target volume was 0.64 ± 1.23 (range 0.01–8.47) cm^3^. The mean lesion margin dose was 14.89 ± 1.90 (range 10–20 Gy). The demographic and clinical characteristics of the overall cohort are detailed in Table 1.

**Table 1:** Comparison of patient’s demographics, clinical variables, and lesion locations (gy: Gray, CM: cavernous malformation, GKRS: Gamma Knife Radiosurgery, DVA: developmental venous anomaly)

		Basal nuclei/ thalamus (*n* = 15)	Brainstem (*n* = 16)	Cerebellum (*n* = 23)	Lobar region (*n* = 80)	All patients (*n* = 134)
Age (years)	Mean ± SD	48.80 ± 13.40	49.94 ± 17.88	49.96 ± 17.75	44.74 ± 15.88	46.71 ± 15.86
	Median (min-max)	54 (18–62)	48,50 (13–76)	51 (23–78)	44,50 (12–81)	47 (12–81)
Gender	Female	9 (60%)	9 (56.3%)	12 (52.2%)	49 (61.3%)	79 (59%)
	Male	6 (40%)	7 (43.8%)	11 (47.8%)	31 (38.8%)	55 (41%)
Follow-up (months)	Mean ± SD	35.53 ± 25.81	46.75 ± 24.49	31.30 ± 23.63	34.56 ± 22.67	35.57 ± 23.54
	Median (min-max)	36 (5–81)	50,50 (2–81)	24 (2–76)	35,50 (2–83)	37,50 (2–83)
Radiation dose (Gy)	Mean ± SD	14.80 ± 2.15	12.94 ± 2.02	14.83 ± 1.47	15.31 ± 1.72	14.89 ± 1.90
	Median (min-max)	14 (12–20)	12 (10–18)	14 (12–18)	14,50 (13–20)	14 (10–20)
Lesion size (cm^3^)	Mean ± SD	1.48 ± 2.41	0.44 ± 0.52	0.45 ± 0.48	0.58 ± 1.12	0.64 ± 1.23
	Median (min-max)	0,31 (0.03–8.32)	0,15 (0.04–1.82)	0,36 (0.01–1.97)	0,29 (0.02–8.57)	0,29 (0.01–8.57)
Surgical intervention before GKRS	Yes	0	1 (6.3%)	0	1 (1.3%)	2 (1.4%)
Hemorrhage before GKRS	No	5 (33.3%)	8 (50%)	16 (69.6%)	51 (63.8%)	80 (59.7%)
	Yes	10 (66.7%)	8 (50%)	7 (30.4%)	29 (36.3%)	54 (40.3%)
Number of hemorrhages	1	8 (80%)	7 (87.5%)	7 (100%)	29 (100%)	51 (94.4%)
	2	2 (20%)	1 (12.5%)	0	0	3 (5.6%)
CM type	Intrinsic	6 (17.1%)	6 (17.1%)	4 (11.4%)	19 (54.3%)	35 (26.1%)
	Exophytic	9 (9.1%)	10 (10.1%)	19 (19.2%)	61 (61.6%)	99 (73.9%)
Zabramski classification	Grade 1	5 (16.1%)	5 (16.1%)	4 (12.9%)	17 (54.8%)	31 (%23.1)
	Grade 2	10 (10.4%)	11 (11.5%)	16 (16.7%)	59 (61.5%)	96 (%71.6)
	Grade 3	0	0	3 (42.9%)	4 (57.1%)	7 (%5.2)
DVA co-existence	No	5 (8.8%)	10 (17.5%)	8 (14%)	34 (59.6%)	57 (%42.5)
	Yes	10 (13%)	6 (7.8%)	15 (19.5%)	46 (59.7%)	77 (%57.5)

### Lesion locations, symptoms, and symptom relation with hemorrhage

Among the patients, 80 (59.7%) had CM in the lobar region, 23 (17.1%) in the cerebellum, 16 (12%) in the brainstem, and 15 (11.2%) in the basal nuclei/thalamus. (Table 1) The most common presentation was headache in 76 (56.7%) patients, followed by imbalance in 41 (30.6%), focal neurological deficit in 17 (12.7%), seizure in 11 (8.2%), nausea in 11 (8.2%) and decrease level of consciousness in 9 patients (6.7%). All patients with decreased consciousness presented with recent hemorrhages and were admitted for observation before GKRS to ensure stabilization without further neurological deterioration. The lesions in patients who presented with headaches and seizures mainly were in the lobar region (78.9% and 81.8%, respectively). In patients with imbalance, CMs primarily were in the cerebellum (19 patients, 46.3%). In patients with focal neurological deficits, CMs were commonly located in the brainstem (52.9%), followed by basal nuclei/thalamus (23.5%). Patients with a decreased level of consciousness predominantly had lesions in the basal nuclei region (44.5%). (Table 2) Among the CMs in 54 patients with hemorrhage, 29 (53.7%) were in the lobar region, 7 (13%) in the cerebellum, 8 (14.8%) in the brainstem, and 10 (18.5%) were in the basal nuclei/thalamus.

**Table 2:** Symptoms according to lesion location and changes after GKRS (GKRS: Gamma Knife Radiosurgery)

		Basal nuclei/thalamus (*n* = 15)	Brainstem (*n* = 16)	Cerebellum (*n* = 23)	Lobar region (*n* = 80)	All patients (*n* = 134)
Symptoms	Headache	5 (6.6%)	3 (3.9%)	8 (10.5%)	60 (78.9%)	76 (56.7%)
	Imbalance	4 (9.8%)	6 (14.6%)	19 (46.3%)	12 (29.3%)	41 (30.6%)
	Decrease in level of consciousness	4 (44.5%)	2 (22.2%)	2 (22.2%)	1 (11.1%)	9 (6.7%)
	Neurological deficit	4 (23.5%)	9 (52.9%)	1 (5.9%)	3 (17.6%)	17 (12.7%)
	Vomiting-nausea	5 (45.5%)	1 (9%)	0	5(45.5%)	11 (8.2%)
	Seizure	1 (9.1%)	1 (9.1%)	0	9 (81.8%)	11 (8.2%)
Engel Seizure classification	1	0	1 (20%)	0	4 (80%)	5 (45.5%)
	3	0	0	0	2 (100%)	2 (18.2%)
	4	1 (25%)	0	0	3 (75%)	4 (36.4%)
Symptom alterations following GKRS	Complete recovery	11 (11.6%)	13 (13.7%)	17 (17.9%)	54 (56.8%)	95 (70.9%)
	No changes	1 (9.1%)	1 (9.1%)	4 (36.4%)	5 (45.5%)	11 (8.2%)
	Worsen	2 (20%)	2 (20%)	0	6 (60%)	10 (7.5%)
	Partial Recovery	1 (5.6%)	0	2 (11.1%)	15 (83.3%)	18 (13.4%)

### Alterations in symptoms following GKRS

The symptoms of 134 patients were evaluated after GKRS according to the lesion’s location. Symptoms of 95 patients (70.9%) were recovered entirely, and in 18 patients (13.4%), symptoms were partially recovered. The complete recovery rate of the symptoms caused by lesions in the brainstem and basal nuclei/thalamus were 81.3% and 73.3%, respectively.

The complete and partial recovery rates for patients with headaches were 68.4% and 18.4%, respectively. The complete and partial recovery rates for patients with imbalance were 75.6% and 12.2%. Following GKRS, focal neurological deficits were totally or partially recovered in 76.5% and 5.9% of the patients, respectively; however, one patient represented increased double vision and imbalance, while another patient developed numbness in the right arm following GKRS. Additionally, among the patients admitted with a change in consciousness, the total recovery rate was 77.8% after a follow-up period. The observed improvement in these patients may broadly reflect the natural progression of the disease, including hemorrhage resorption and resolution of cerebral edema, rather than the therapeutic effect of GKRS. The seizure control was achieved at Engel Class 1 for five patients (45.5%) and Engel Class 3 for two patients (18.2%). However, one patient experienced an increased seizure rate after GKRS. The patients’ symptoms and symptom alterations after GKRS were summarized in Table 2.

### Hemorrhage rates and evaluation of factors in post-GKRS hemorrhage

Before undergoing GKRS, patients were observed for an average period of 7.16 ± 13.21 months (range: 1–86 months), resulting in a cumulative total of 79.92 patient-years. During this period, 57 hemorrhagic events were detected in 54 patients (Table 1). However, when excluding patients who presented with hemorrhages at their initial admission, the number of hemorrhages identified during the follow-up period was five. The AHR was calculated to be 6.26%. Two of these five hemorrhages were initial occurrences, and three were recurrent. Another approach to these calculations would be to consider the congenital nature of these lesions and start the observation period at birth. If the observation period is extended from the birth of the patients, the resulting AHR is 0.91% (57 hemorrhages over 6259 patient-years).

Across all patients, the total follow-up duration after GKRS was 397.17 patient-years. During this period, hemorrhage occurred in four patients, resulting in an AHR of 1.01%. In the first 2 years, there were 209.8 years of observation and 3 hemorrhages during this period; this led to an annual post-GKRS hemorrhage rate of 1.43% during the first 2 years after treatment. For the remaining follow-up period, there was 1 hemorrhage during 187.3 patient-years of observation. These data resulted in an annual post-GKRS hemorrhage rate of 0.53% after 2 years of follow-up.

This calculations, derived from a cohort of symptomatic individuals, does not account for the broader population of asymptomatic CMs, which may never bleed or remain undiagnosed. The inclusion of such cases would significantly dilute the “never-bled” subjects and further lower the natural AHR, underscoring the limitations and complexities in estimating AHR.

The pre-treatment observation period for patients with a history of hemorrhage totaled 46.6 patient-years, during which 57 hemorrhages were recorded. After excluding the first 54 hemorrhages, AHR was calculated at 6.44%.

In a subgroup of patients that experienced previous hemorrhage, following GKRS, four hemorrhages occurred over a follow-up duration of 196.3 patient-years, resulting in a post-GKRS AHR of 2.04%. Three hemorrhages were observed during the first two years post-GKRS, corresponding to an AHR of 3.05%. Beyond the initial two-year latency period, the AHR decreased to 1.02%, with only one bleeding reported in the subsequent follow-up.

There was no significant association between post-GKRS hemorrhage and gender (*p* = 0.179), age (*p* = 0.787), Zabramksi Grade (*p* = 0.148), being exophytic or intrinsic (*p* = 0.660), or DVA coexistence (*p* = 0.492). However, the volume of the CM was found to be a statistically significant risk factor for rebleeding (*p* = 0.035). The most critical risk factor for subsequent hemorrhage after GKRS was a prior hemorrhage (*p* = 0.025). After GKRS, patients with rebleeding were in the lobar area (2/80, 2.5%) and the basal nuclei/thalamus (2/13, 18.2%). No rebleeding was observed in other locations. The rebleeding rate in lesions in the basal nuclei/thalamus was statistically significantly higher (*P* = 0.045).

### Adverse radiation effects

**Fig. 1:**
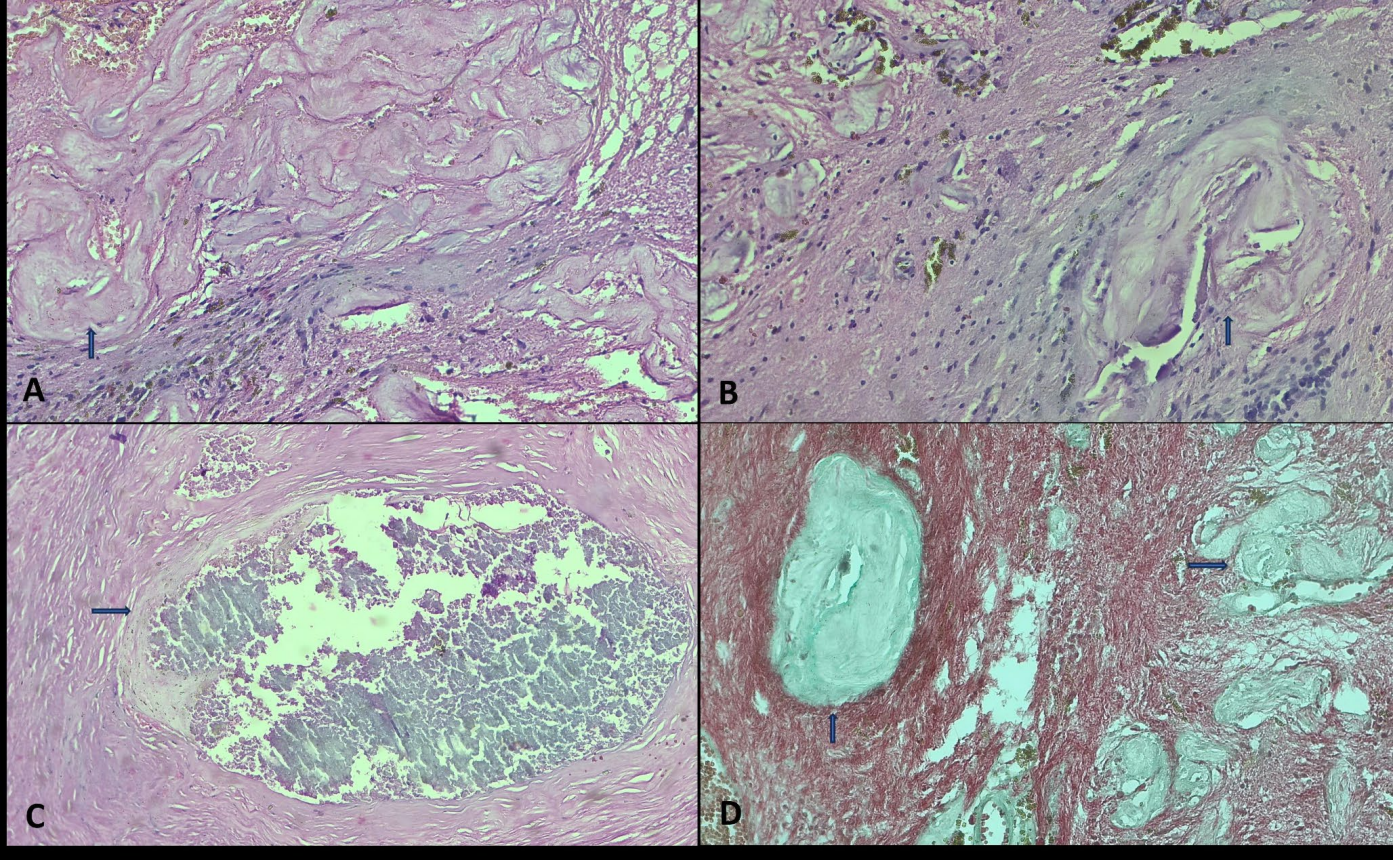
**A**: A 44-year-old male patient underwent GKRS (13 Gy) due to a hemorrhagic CM in the right mesencephalon. **B**: The patient’s diffusion MRI at the fourteenth month showed edema at the level of the crus cerebri, and the patient’s complaint of diplopia became permanent. **C**: A 24-year-old male patient underwent GKRS (15 Gy) due to a CM at the left centrum semiovale. **D**: The patient’s computed tomography at six months revealed perilesional edema. The dysesthesia in the patient’s right arm became permanent during follow-ups

**Fig. 2:**
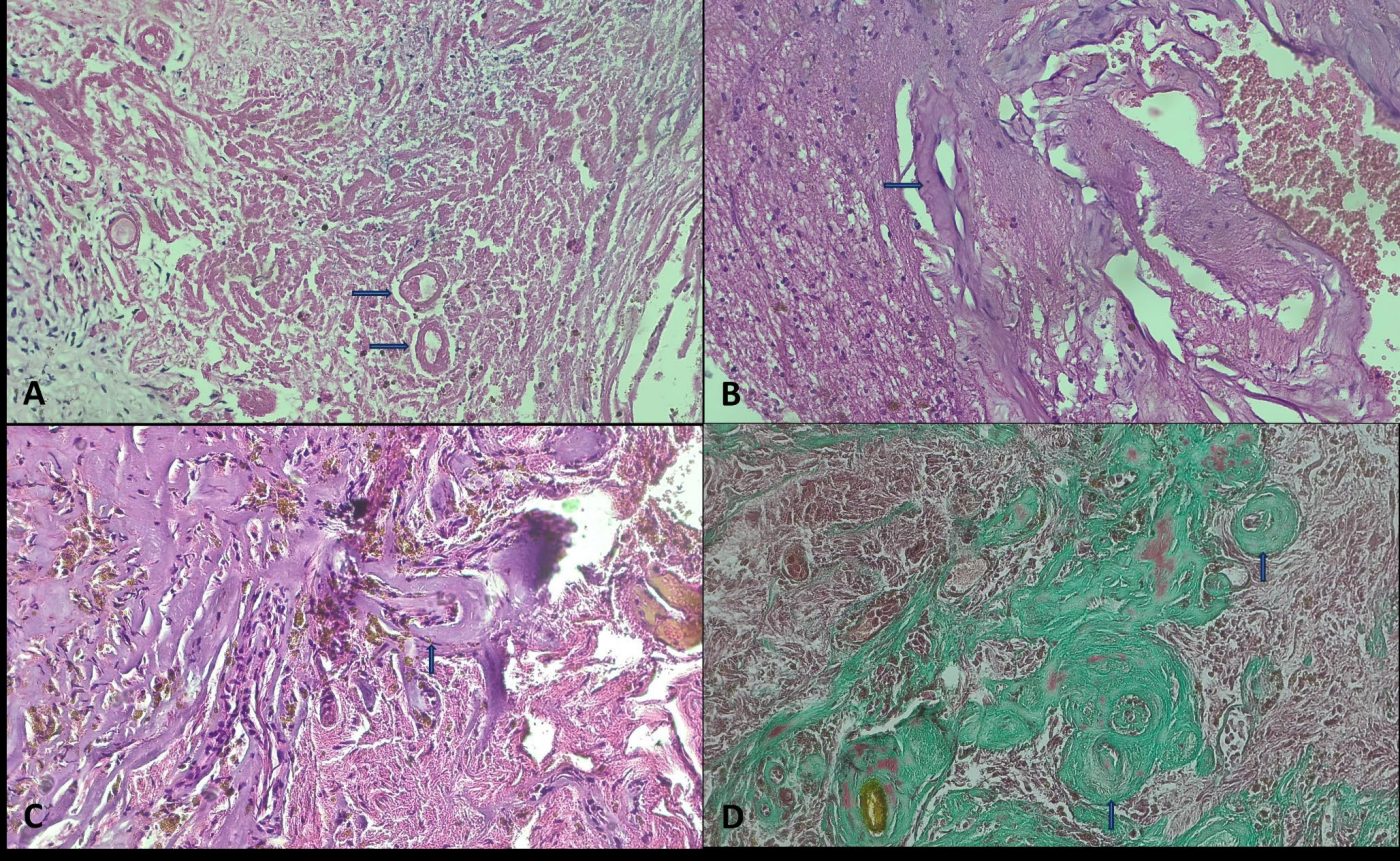
**A**: A 60-year-old male patient was investigated due to a history of seizures, which revealed a right frontal CM, and underwent GKRS (14 Gy). **B**: The twelfth-month follow-up MRI showed perilesional edema. **C**: The patient underwent surgery due to intractable seizures, resulting in seizure control. The histopathological changes are given in Fig. 3

**Fig. 3:**
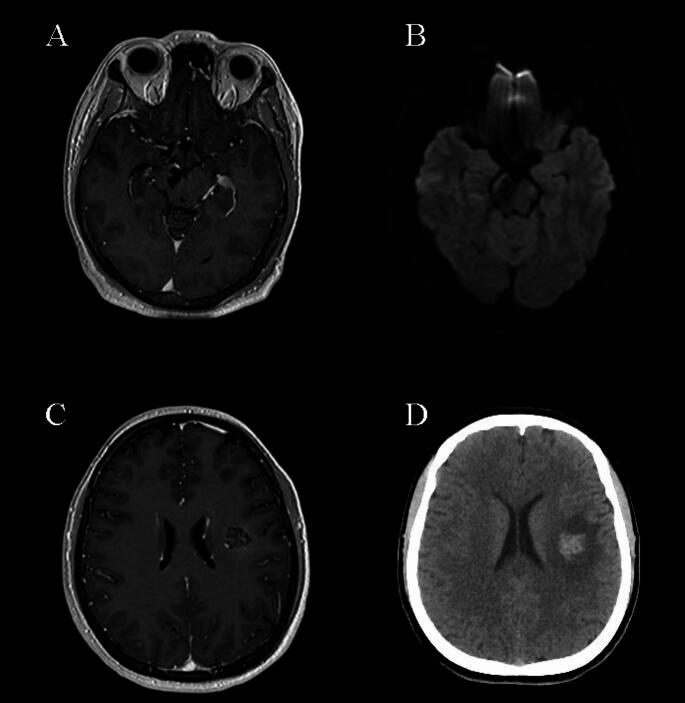
The patient reported in Fig. 2 underwent histopathological evaluation. **A**, **B**: Tissue sections show multiple sclerotic vascular walls and narrowed vessels (arrows). Hematoxylin Eosin. Original magnification X100. **C**: Intraluminal thrombus occurred after GKRS, as shown by the arrow. Hematoxylin&Eosin. Original magnification X100 **D**: Multiple sclerotic vascular walls and narrowed vessels (arrows) Masson Trichrome, Original magnification ×200

**Fig. 4:**
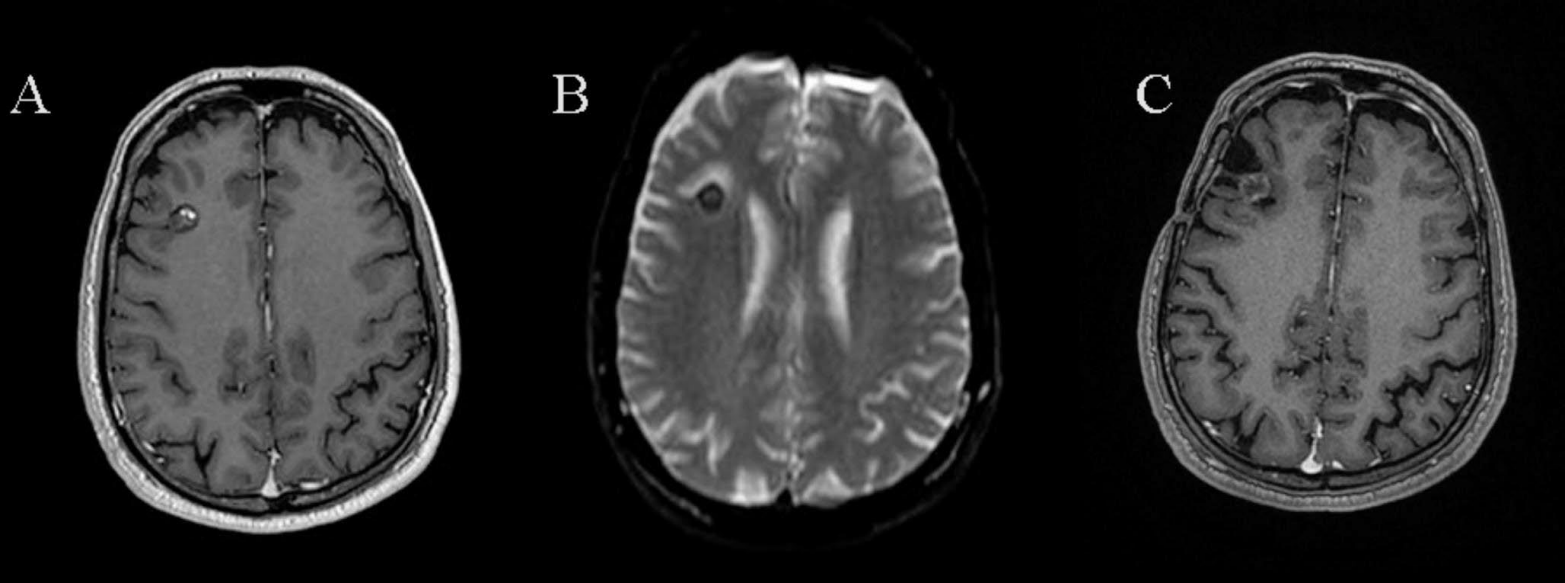
A CM is surgically removed two years after GKRS because of severe headache and underwent histopathological evaluation **A**: Tissue sections show multiple vascular structures with hyalinization and fibrinoid necrosis (arrows). Hematoxylin Eosin. Original magnification X200 **B**, **C**: Vascular structures with multiple luminal narrowing and sclerosis (arrows). Hematoxylin Eosin. Original magnification X200 **D**: Multiple sclerotic vascular walls and narrowed vessels (arrows) Masson Trichrome, Original magnification ×200

Overall, ten patients (7.46%) had perilesional edema following GKRS. Among them, three patients (2.2%) had permanent deficits: one patient had double vision (Fig. 1A-B), one had dysesthesia in his right hand (Fig. 1C-D), and one patient had an increase in his seizure frequency (Figs. 2 and 3). For the patient with an increased seizure rate, which unresponsive to medical treatment, surgery was performed one year after GKRS, and seizure control was achieved as grade 2 according to the Engel Classification System. Another patient suffering from severe headache, unresponsive to medical treatment, underwent surgery two years after GKRS (Fig. 4), resulting in the resolution of the severe headache. The patients with permanent double vision and dysesthesia were not subjected to surgery. The remaining six patients (4.5%) showed worsening headaches with perilesional edema but were relieved with medical treatment during the follow-up period.

### Histopathologic evaluation of CMs following GKRS

Surgically resected lesions underwent histopathological examination. In tissue sections, numerous vascular structures have sclerotic vessel walls, luminal narrowing due to hyalinization, fibrinoid necrosis, and intraluminal thrombus caused by radiation. The histopathological evaluations of the patients who underwent GKRS are shown in Figs. 3 and 4.

## Discussion

There are ongoing debates regarding assessing hemorrhage risk due to the imprecise knowledge concerning the duration of a CM’s presence in a patient [9, 35]. Since it is not feasible to observe patients with CMs throughout their entire lifespan before and after GKRS, the exact ratio of annual hemorrhage frequency (AHF) before and after GKRS cannot be determined. However, various calculation methods have been employed in the literature and our study to evaluate the effect of GKRS with the best possible accuracy. Some calculations assume the lesions have been present since birth, while others are based on detecting the first hemorrhage or diagnosis.

If we assume that CMs are congenital and that there were no hemorrhages until the patient was first diagnosed, the AHR may appear lower than it actually is. The follow-up periods after GKRS will be relatively short compared to the time elapsed from birth. This may lead to the number of hemorrhages observed in the post-GKRS period being disproportionately high relative to the follow-up duration, causing the effect of GKRS on AHR to appear less significant.

When we calculate AHRs starting from the time of the initial presentation, it should be noted that 40% of the patients had already experienced a hemorrhage at their first presentation in our series. Since re-hemorrhage is more likely after an initial bleed, the hemorrhage rates during this period may appear higher than the actual rates in the total CM population. Consequently, this calculation could overestimate the protective effect of GKRS.

Moreover, our cohort of never-bled patients consists exclusively of individuals who sought medical attention at our institute, potentially overlooking a vast population of asymptomatic or undiagnosed CMs. This broader, often-unrecognized group implies that the true natural AHR for never-bled CMs may be substantially lower, as many individuals may never experience hemorrhage or even become aware of their lesion’s existence. Such population-level considerations further complicate efforts to define a universal AHR and highlight that this debate will likely remain unresolved.

As it is well known, the protective effect of GKRS becomes more apparent after the 2-3-year latency period. Therefore, after initially calculating the total observation period following GKRS, we divided it into two phases: 0–2 years and the period thereafter. This division aimed to assess the effect of GKRS separately during the latency period and the post-latency period.

Despite these varied methods, studies have consistently indicated that the rate of additional hemorrhages decreases following GKRS treatment. According to Kondziolka et al., the AHR decreased from 32 to 8.8% in the first two years after the procedure and further reduced to 1.1% during the 2 to 6-year interval post-radiosurgery [22]. Similarly, Chang et al. observed a decrease from 9.4 to 1.6 after 36 months, and Kim et al. documented a reduced AHR from 35.5 to 3.55% after GKRS [5, 21]. Our study confirms that GKRS significantly reduces the risk of subsequent hemorrhages particularly in previously bled CMs, providing strong justification for its utilization in these cases. This protective effect was evident across all calculation approaches and aligns with existing studies demonstrating its efficacy. However, the inclusion of a broader, asymptomatic population could potentially affect the natural AHR, highlighting the complexity and subjectivity of these calculations. Moreover, given the robust literature and our own findings demonstrating a marked reduction in recurrent hemorrhages in previously bled CMs, GKRS emerges as a justified and favorable treatment option for these cases.

The mechanism of the radiobiological effect of GKRS in reducing the hemorrhage rates in CM is still in debate [29, 43]. Mainly, volume changes and obliteration of the vascular channels cannot be demonstrated with conventional imaging methods [42]. However, Hasegawa et al. proposed a hypothesis suggesting that radiation-induced angiopathy leads to the obliteration of microvascular structures within lesion lumens [15]. Gewirtz et al. histopathologically evaluated lesions surgically excised after helium ion radiation therapy, revealing a combination of vessel fibrosis, fibrinoid necrosis, and ferrugination [11]. Similarly, Shin et al. analyzed CM samples post-GKRS in various resection periods, observing vascular sclerosis in all lesions. Partial narrowing was observed in lesions resected four months after GKRS, while most vessels showed obliteration in lesions resected three and seven years post-GKRS [44]. Additionally, Tu et al. demonstrated partial occlusion and proteinaceous thrombin in CMs after linear accelerator radiosurgery [46]. In our study, histopathological evaluation of surgical specimens obtained one and two years after GKRS demonstrated numerous vascular structures with luminal narrowing, hyalinization, and fibrinoid necrosis. Nonetheless, histopathological samples also revealed the presence of patent vessels, potentially leading to hemorrhage post-GKRS.

Although the primary focus in GKRS is to reduce the risk of bleeding, it is crucial to prioritize symptoms and neurological changes in patients, irrespective of radiologically documented hemorrhage [13, 35]. Notably, the absence of further hemorrhages on imaging does not exclude the possibility of new or persistent neurological symptoms, underscoring that no detectable new bleeding does not necessarily equate to a complete resolution of clinical problems.

In their research, Lee et al. showed improvement or stabilization of the symptoms in 95.9% of their patients [25]. Monaco et al. reported similar outcomes, with 79.4% of their patients experiencing improvement or stabilization in symptoms, particularly for lesions located in the brainstem [31]. In our study, we found that for lesions located in the brainstem or basal nuclei/thalamus, total or partial recovery was achieved at a rate of 80.6%. Similarly, for symptoms caused by lesions in the cerebellum and lobar region, the rate of total or partial recovery was 85.4%. However, as in this study, the retrospective data collection poses significant methodological challenges. Symptoms such as headaches and imbalance were documented using non-standardized criteria, introducing variability and potential biases in the assessment of outcomes. The observed improvement rates for these subjective symptoms may partially reflect the natural course of the disease rather than the therapeutic effects of GKRS. Consequently, these findings should be interpreted cautiously, given the reliance on non-uniform medical records and inconsistent symptom reporting. Employing standardized assessment tools and validated scoring systems is essential for enhancing the precision and reliability of future investigations.

Kondziolka et al. [23] conducted a retrospective study involving a group of 122 patients managed conservatively and had an AHR of 1.3%. Over a 34-month follow-up period, nine patients experienced a new hemorrhage, and six developed new neurological deficits. For patients without a prior bleed, the annual rate was 0.6%, while those with a previous hemorrhage had a rate of 4.5%. In a recent study [40] involving a symptomatic group of 265 patients, 131 (49%) had a history of symptomatic hemorrhage at baseline. In this patient group conservatively managed, during a median follow-up of 58 months, 51 patients (19%) experienced symptomatic hemorrhages, 33 (12%) had seizures, and 13 (5%) developed focal neurological deficits, with 14 patients (5%) undergoing surgical or radiosurgical interventions and two (0.8%) dying from CM-related causes. In contrast, our series of 134 patients treated with GKRS revealed only four cases of rebleeding. While current literature cites a 19% symptomatic hemorrhage rate when conservatively managed, our findings show a significantly lower rate of 2.9% (4/134) after GKRS. Additionally, a recent multicenter cohort study [24] with a 5-year follow-up focused solely on brainstem lesions compared conservative management and radiosurgery. As a result, a lower hemorrhage rate was reported in the radiosurgery group. These results suggest that GKRS could be a more favorable approach compared to conservative management.

An analysis of seizure control in 291 patients across 16 studies revealed that complete seizure control was achieved in 31% of patients. In contrast, a decrease in seizure frequency was observed in 35% of patients after GKRS [33]. In our research, Engel Class I and II control was achieved in 45.5% of the patients with seizures. Notably, one patient experienced an increase in seizure frequency and underwent surgical resection. However, in significant microsurgical series, total seizure control was reported at 82.9%, 80.3%, and 72.4% [4, 5, 7, 31]. Since there is a negative correlation between epilepsy duration and seizure control after intervention and a time interval is needed to observe the effect of GKRS, the success rate of microsurgery in seizure control is higher, as expected [38].

GKRS is an effective treatment method for relieving symptoms and reducing hemorrhage in CMs. Our study’s complete and partial recovery rates for headaches were 68.4% and 18.4%, respectively. While we cannot definitively attribute the recovery of this subjective symptom to GKRS, headaches remain an essential symptom that can significantly affect patients’ quality of life and represent an area requiring further research to understand their mechanisms and management better. In contrast, the improvements observed in other symptoms, such as imbalance and neurological deficits, have been attributed to the effects of radiosurgery.

However, AREs remain a recognized concern with GKRS. In a meta-analysis of 8 reports, cyst formation, edema, and neurological deficits were the most common complications encountered after GKRS [47]. Current meta-analyses report a permanent AREs rate of 4% [43]. Especially in the early use of GKRS, high rates of neurological complications were observed [19, 27, 34, 47]. However, these complication rates were correlated with higher marginal doses (> 13 Gy) and high volumes (> 0.7 cc) [6, 10]. In a study involving 298 patients across different GKRS centers in Japan, complications were found more frequent for lesions bigger than 15 mm in size and when the marginal dose exceeded 15 Gy. Their study found that the complication rate was 10% when the marginal dose exceeded 15 Gy [20]. Similarly, Monaco and colleagues [31] reported that the rate of new neurological deficit was 11.8%. Importantly, in both studies, these complications were mostly transient. Younger patients below 30 years of age and patients with a prior surgical intervention are also considered at-risk groups for neurological worsening [6, 28]. In our series, the decision to treat patients younger than 30 with GKRS was based on several factors, including multiple hemorrhages, symptomatic presentations corresponding to the CM location, and patient preferences after detailed counseling about the risks and benefits of treatment. For non-hemorrhagic cases in this age group, the rationale for GKRS was to address significant symptoms such as seizures or focal neurological deficits that were refractory to medical management, particularly in patients with deep-seated lesions or those located in eloquent areas where surgical intervention posed significant risks. Furthermore, besides dose and target, preserving a DVA if it coexists with CM is essential in reducing the complication rate [41]. Additionally, the finding that the presence of DVA increases the risk of hemorrhage in some studies emerges as an exciting observation [6]. In our study, the rate of AREs was 7.46%, and only 3 patients experienced new permanent neurological deficits.

### Limitations

The main limitations of our study are its single-center and retrospective design. Including two patients who had undergone prior surgical interventions and one treated with GKRS at another center introduces potential biases, affecting the generalizability of our findings. Differences in treatment protocols and patient management between centers could also contribute to outcome variability, such as symptom recovery and hemorrhage rates. However, including these patients was necessary due to residual lesions in the operated cases and the availability of complete treatment data for those treated elsewhere.

The absence of a strictly enforced minimum follow-up duration introduces variability in the timing of clinical and radiological assessments. While this approach allowed us to capture outcomes during the latent period, it may affect the evaluation of long-term results. The lack of a comparative group of CMs managed conservatively without GKRS further limits our ability to assess the relative efficacy and safety of GKRS directly.

The limited number of cases that underwent surgery following GKRS restricts the generalizability of histopathological findings and their implications for symptom relief and hemorrhage reduction.

Lastly, our study could not fully standardize complaints such as imbalance, headache, and similar symptoms. As with most retrospective research, non-uniform or lacking standardized data acquisition represents a notable limitation.

## Conclusions

Our study demonstrates that GKRS is an effective treatment method for reducing AHR and relieving symptoms in patients with symptomatic CMs with a low complication rate. While most clinical studies rely on clinical observation to understand the effects of GKRS, histopathological changes following GKRS reveal possible mechanisms for reducing hemorrhage risk and symptoms. Although our study does not directly compare GKRS with observation, the results suggest that GKRS may benefit patients with CMs. Therefore, while GKRS remains a promising option for managing symptomatic CMs, clinicians should recognize that it is not without risks, and longer-term follow-up is essential to confirm its safety and efficacy. A careful, individualized approach to patient selection and ongoing monitoring is warranted to optimize outcomes.

## Data Availability

No datasets were generated or analysed during the current study.
